# SET-HOI: Skeleton-Enhanced Transformer with Parallel Fusion for HOI Detection

**DOI:** 10.3390/biomimetics11100687

**Published:** 2026-09-23

**Authors:** Rui Xiong, Bohong Wu, Qing Gao

**Affiliations:** School of Electronics and Communication Engineering, Sun Yat-sen University, Shenzhen 518107, China; xiongr9@mail2.sysu.edu.cn (R.X.); wubh6@mail2.sysu.edu.cn (B.W.)

**Keywords:** human–object interaction (HOI), graph convolutional network (GCN), skeleton

## Abstract

Human–Object Interaction (HOI) detection seeks to identify the relationships between humans and objects in images that play a pivotal role in high-level vision tasks and biomimetic systems, such as biomimetic robotics, intelligent prosthetics, and embodied AI for intent understanding. However, existing approaches that rely solely on visual appearance features are susceptible to background interference, which adversely affects detection accuracy. Inspired by the biomimetic dual-stream mechanism of biological visual perception, we propose a Transformer-based HOI detection model with a dual-branch parallel fusion architecture, incorporating a skeleton topology branch alongside the standard image branch. The skeleton branch leverages a Graph Convolutional Network (GCN) to explicitly model spatial–kinematic relationships between human keypoints and objects, providing fine-grained topological priors to complement visual appearance features before joint decoding. Our approach achieves 51.6% Mean Average Precision (mAP) on the Verbs in Common Objects in Context (V-COCO) dataset, outperforming the Transformer-based HOI baseline by 2.7 mAP points, highlighting its effectiveness in enhancing perceptual robustness for biomimetic interactive applications.

## 1. Introduction

Human–Object Interaction (HOI) detection can be regarded as an extension of the object-detection task [1]. Its goal is not only to detect humans and objects in an image but also to classify the dynamic interactions between them, as shown in Figure 1. Beyond visual understanding, HOI detection plays a fundamental role in modern biomimetic systems, such as biomimetic robots, intelligent prosthetics, and embodied artificial intelligence (AI), by enabling these systems to comprehend human behavioral intent, anticipate interactive actions, and perform safe human–robot collaboration. Inspired by how biological cognitive systems disentangle visual appearance from spatial–motor dynamics, computational HOI approaches can be broadly categorized into two-stage and one-stage methods. Two-stage methods [2] first detect humans and objects separately and then determine their interactions based on spatial and contextual information. In contrast, one-stage methods directly predict 〈human,object,interaction〉 triplets from global image features, thereby exploiting holistic context [3].

The earliest HOI detection approaches were largely based on the two-stage pipeline, which offered relatively high accuracy. However, due to their limited model integration and low efficiency, research attention gradually shifted toward one-stage approaches, which significantly improves efficiency by leveraging global image information. Recently, Transformer-based one-stage HOI detection methods have emerged as the dominant paradigm. Inspired by DETR [5], these methods treat HOI detection as a set prediction problem, producing 〈human,object,interaction〉 triplets in an end-to-end manner. This eliminates the need for non-maximum suppression (NMS) [6] and avoids complex hand-crafted post-processing, resulting in a more efficient and concise inference pipeline.

Nevertheless, Transformer-based end-to-end methods rely heavily on the representational capacity of Transformer to capture visual features from raw RGB images [7]. Such models are trained to implicitly attend to action-relevant regions through large-scale data. However, image-only information often has inherent limitations. Due to constraints in camera viewpoints, issues such as blurred boundaries of human or objects and severe occlusions frequently occur. Unlike artificial visual systems that struggle in these ambiguous conditions, biological perception leverages spatial–kinematic priors to explicitly model topological skeletal structure and object positions. Under degraded visual conditions, although object-detection and pose-estimation models can perform well [8], HOI detection models that depend solely on visual features have difficulty capturing fine-grained spatial relations between human and objects [9], and they lack explicit modeling of human–object topological structures. Consequently, their performance may degrade in complex interaction or occlusion scenarios.

To address these challenges, inspired by the biomimetic dual-stream mechanism of biological cognitive systems, we propose an enhanced HOI detection framework based on Transformer, aiming to more accurately localize human and objects and structurally model their interactions. Specifically, we design a dual-branch Skeleton-Transformer HOI (SET-HOI) model consisting of an image branch and a skeleton branch, mirroring the ventral (semantic appearance) and dorsal (spatial–kinematic topology) streams of the human brain. The image branch retains the conventional backbone + Transformer + Multi-Layer Perceptron (MLP) pipeline. The skeleton branch, on the other hand, first predicts human skeleton and object positions, then constructs topological graphs using skeleton keypoints and object locations. These graphs are processed with a Graph Convolutional Network (GCN) [10,11] to extract interaction-related features, which are subsequently encoded into a global memory representation. The global memory from the skeleton branch is fused with that from the image branch and passed into the Transformer decoder, which outputs vectors that represent human–object interactions. Finally, a Multi-Layer Perceptron (MLP) parses these vectors to obtain all HOI triplets in the image.

The main contributions of this work are threefold. First, bridging biomimetic perception and interactive applications, we introduce a novel dual-encoder architecture, termed SET-HOI, which separately processes image and skeleton modalities to capture heterogeneous feature representations. Second, by incorporating a Graph Convolutional Network (GCN), human–object interaction graphs are explicitly constructed using both skeleton keypoints and object positions, mimicking the biological modeling of spatial and semantic topological relations to provide richer perceptual priors for biomimetic interactive devices. Finally, our framework achieves a more balanced and efficient multimodal fusion by integrating the refined skeleton-GCN features with the image branch at the decoder stage.

Since HOI detection was first introduced in 2015, a wide range of solutions have been proposed. Early methods adopted two-stage frameworks [12], which separately performed object detection and interaction recognition. Over time, research has shifted toward end-to-end one-stage approaches. Our work builds upon such end-to-end HOI models by introducing a skeleton branch to enable more fine-grained interaction reasoning.

One-stage HOI detection with Transformers is inspired by Detection Transformer (DETR) [5], which reformulates object detection as a set prediction problem. By employing a Transformer encoder–decoder architecture and bipartite matching loss, DETR eliminates the need for NMS and achieves true end-to-end detection. In 2021, Zou et al. [4] first explored applying Transformer encoders to HOI tasks, leveraging their strong contextual modeling capacity to capture human–object relations, and laid the foundation for a CNN–Transformer–MLP hybrid HOI framework. In the same year, Tamura et al. [13] fully adapted the DETR paradigm to HOI detection by introducing learnable interaction queries, each responsible for predicting a triplet. Through the decoder’s attention mechanism, each query automatically attends to 〈human,object〉 regions that are likely to involve interactions. Kim et al. proposed HOTR [14], which also builds on DETR but uses a shared encoder and two separate decoders for human–object and interactions, later linking them via interaction points to form triplets.

In recent years, inspired by generative language models, some researchers have begun exploring approaches that leverage generative methods combined with large pre-trained models to extract human interaction information from images [15,16]. Although these approaches effectively exploit Transformer-based visual modeling, they remain constrained by their reliance on pure image features. To address this, Yue et al. proposed Guided-Embedding Network with Visual-Linguistic Knowledge Transfer (GEN-VLKT) [17], which enhances generalization by integrating large-scale prior knowledge from vision-language models. GEN-VLKT combines generative verb prediction with knowledge transfer from Contrastive Language-Image Pretraining (CLIP) [18]. Specifically, it converts verb category names into textual embeddings via CLIP’s text encoder as “targets”, while a Transformer decoder generates verb semantics as “predictions”. By matching generated and textual features, the model reformulates verb classification in HOI as a feature generation and alignment task. While innovative, this approach still depends on CLIP’s global semantic priors, which may become unreliable under occlusion, leading to missed or incorrect detections. Motivated by this limitation, we aim to explicitly introduce human skeleton and object positional information, which can compensate for occluded regions and provide more complete cues for HOI reasoning.

Skeleton representations have achieved significant progress in action recognition and pose estimation, and have been widely applied across vision tasks. In action recognition, Zhang et al. proposed SA-GCN [19], which integrates self-attention into GCN architectures to enhance multi-scale joint dependency modeling. More recently, PCCTR-GCN [20] was proposed to improve GCN adaptability to joint relations by introducing pose correction and channel topology refinement, yielding notable accuracy gains. Skeleton information has also been exploited in pose estimation. For example, Rantelinggi et al. [21] demonstrated skeleton estimation using Frequency Modulated Continuous Wave (FMCW) radar, showing its effectiveness in complex environments. Furthermore, advancements in 3D pose estimation [22,23,24,25] provide strategies to address challenges such as occlusion, cross-view generalization, and data scarcity, reinforcing the theoretical foundation of skeleton-based representations.

In the context of HOI detection, skeleton information is naturally correlated with object interactions—for instance, a hand near a cup often implies holding or pouring. This motivates the use of graph-based models to capture spatial dependencies between humans and objects. Qi et al. proposed Graph Parsing Neural Network (GPNN) [26], which combines graph reasoning with neural representation learning in an end-to-end framework to model dynamic human–object relations. Although effective in structural reasoning, GPNN relies on multi-round message passing, which introduces inference latency, and lacks the modeling power of self-attention, limiting its scalability to large datasets and diverse interactions.

In contrast, our method leverages the expressive reasoning capacity of Transformer while explicitly incorporating skeleton–object structural information. This fusion not only improves performance under occlusion and complex interactions but also enables explicit modeling of human–object spatial dependencies. As a result, integrating skeleton features complements visual cues and enhances the robustness and accuracy of HOI detection in challenging scenarios.

## 2. Materials and Methods

### 2.1. Model Architecture

The proposed SET-HOI model enhances Transformer-based HOI detection by integrating a skeleton branch to explicitly model human–object structural relations. As shown in Figure 2, the image branch extracts visual features via a CNN backbone and encodes them with a Transformer to capture global context. In parallel, a pose estimator and object detector provide skeletal keypoints and object positions, which are used to construct a relational graph. These graphs are first processed by the GCN to obtain pair-wise structural embeddings, which are subsequently encoded by a Transformer-based skeleton encoder to produce skeleton contextual memory. The global representations from both branches are fused and passed to the Transformer decoder, enabling joint reasoning over visual and structural cues. Finally, an MLP parses the decoded representations into 〈human,object,interaction〉 triplets as the final prediction.

### 2.2. Image Branch

The image branch consists of a CNN backbone for visual feature extraction and a Transformer encoder for global representation learning. During processing, the CNN progressively extracts features through convolution and pooling layers, producing a feature map. A 1 × 1 convolution reduces the channel dimension from C = 2048 to d = 256, yielding a feature representation of size (H × W, d). To match the Transformer’s input format, the feature map is flattened into one-dimensional vectors.

Since positional information is essential for capturing spatial relationships, we compute positional embeddings and combine them with the visual features as follows:

(1) $${\mathrm{Input}}_{t}={\mathrm{Features}}_{t}+{\mathrm{Pos}}_{t},$$

where ${\mathrm{Pos}}_{t}$ denotes the positional embedding associated with the *t*-th element in the sequence.

The Transformer encoder then applies multi-head self-attention to construct inter-feature relationships, dynamically computing attention weights to capture long-range dependencies (e.g., between human limbs and interacted objects). Spatial attention further refines semantic weights, followed by a feed-forward network (FFN [27]) and MLP layers to integrate local details and global semantics. This process yields the global contextual memory of the image branch.

### 2.3. Skeleton Branch

As illustrated in Figure 2, the skeleton branch operates in parallel with the image branch, providing complementary topological and kinematic cues. The input image is first processed by a pose estimator and object detector to obtain 18 human skeletal keypoints and object positions (represented by the diagonal points of bounding boxes), using pre-trained pose-estimation and detection models.

*Skeleton–Object Graph Construction.* Skeletal keypoints are connected according to a fixed human anatomical topology, capturing spatial dependencies among body joints. For each detected human–object candidate pair, the object is represented by two nodes corresponding to the diagonal corner points of its bounding box. These object nodes are incorporated into the skeletal topology to form a human–object interaction graph. To handle scenes with multiple humans and objects while avoiding feature interference across different candidate pairs, we decompose the detected entities into $N_{\mathrm{pairs}}=N_{p}\times N_{o}$ independent human–object subgraphs, where each subgraph corresponds to one candidate pair $\langle {\mathrm{Human}}_{i},{\mathrm{Object}}_{j}\rangle$.

Each subgraph G=(V,E) contains $N_{\mathrm{nodes}}=20$ nodes, consisting of $V_{\mathrm{skel}}=18$ anatomical keypoints and $V_{\mathrm{obj}}=2$ object nodes. A standard 2-layer GCN is employed to extract pair-wise structural features. Message passing is performed using the symmetrically normalized adjacency matrix $\tilde{A}=D^{-\frac{1}{2}}\left(A+I_{20}\right)D^{-\frac{1}{2}}$:

(2) $$H^{\left(l+1\right)}=\mathrm{ReLU}\left(\mathrm{BatchNorm}\left(\tilde{A}H^{\left(l\right)}W^{\left(l\right)}\right)\right).$$

Here, $W^{\left(l\right)}$ denotes the learnable transformation matrix. The hidden dimension is first projected to 128 and then to C=256 to match the Transformer channel dimension, with a dropout rate of 0.1. This lightweight GCN explicitly models the spatial–topological relationships between human joints and the spatial extent of the candidate object while introducing only limited computational overhead.

*Handling Multiple Interaction Pairs.* For scenes containing multiple entities, AlphaPose is used to extract human keypoints and YOLOv8s is used to detect object candidates. Given $N_{p}$ detected humans and $N_{o}$ detected objects, we enumerate all possible human–object combinations, resulting in $N_{\mathrm{pairs}}=N_{p}\times N_{o}$ candidate interaction graphs. Each graph is processed independently by the GCN, as illustrated in Figure 3.

*Skeleton Feature Encoding.* For each candidate human–object subgraph, the GCN produces a *C*-dimensional pair-wise structural embedding $z_{\mathrm{graph}}^{i}\in R^{C}$. The embeddings of all candidate pairs are then organized into a sequence,

(3) $$Z_{\mathrm{graph}}=\left[z_{\mathrm{graph}}^{1},z_{\mathrm{graph}}^{2},\ldots ,z_{\mathrm{graph}}^{N_{\mathrm{pairs}}}\right],\;Z_{\mathrm{graph}}\in R^{N_{\mathrm{pairs}}\times B\times C}.$$

Rather than directly using these local graph embeddings for fusion, $Z_{\mathrm{graph}}$ is further processed by a Transformer-based skeleton encoder, which adopts the same encoder architecture as the image branch. Through multi-head self-attention, the skeleton encoder models contextual dependencies among different candidate human–object pairs and produces the skeleton contextual memory:

(4) $$M_{\mathrm{skel}}={\mathrm{Encoder}}_{\mathrm{skel}}\left(Z_{\mathrm{graph}}\right),\;M_{\mathrm{skel}}\in R^{N_{\mathrm{pairs}}\times B\times C}.$$

This pair-wise memory preserves the structural representation of each candidate interaction while introducing global contextual reasoning across different human–object pairs. It therefore complements the image memory, particularly in scenarios involving human occlusion or ambiguous object boundaries. The subsequent fusion of the skeleton and image memories is illustrated in Figure 4.

### 2.4. Transformer-Based Fusion Module

After obtaining the image contextual memory $M_{\mathrm{img}}$ and the skeleton contextual memory $M_{\mathrm{skel}}$ from their respective Transformer encoders, SET-HOI concatenates the two representations along the sequence dimension:

(5) $$M_{\mathrm{struct}}=\left[M_{\mathrm{img}}\|M_{\mathrm{skel}}\right],$$

which is then fed into the Transformer decoder.

Each decoder layer comprises three components: multi-head self-attention, cross-attention, and an FFN. In the cross-attention layer, the Value is taken from encoder memory, the Key combines global contextual memory with positional encoding, and the Query integrates positional encoding with HOI queries. This design allows each query to focus on the most relevant regions by computing attention weights between queries and global memory. The self-attention layer, in contrast, operates over HOI queries (or the previous decoder output), enabling refinement of query representations and prediction of multiple interactions. Multi-head self-attention further enhances the modeling of diverse interaction patterns.

In the cross-attention layer, the interaction queries $Q\in R^{K\times C}$ perform attention over $M_{\mathrm{struct}}$:

(6) $$H_{\mathrm{dec}}=\mathrm{Softmax}\left(\frac{QW_{Q}{\left(M_{\mathrm{struct}}W_{K}\right)}^{T}}{\sqrt{d_{k}}}\right)M_{\mathrm{struct}}W_{V}.$$

To prevent representation collisions in multi-entity scenarios, global bipartite Hungarian matching is applied during training, ensuring that each query uniquely grounds one specific human–object pair.

The decoder adaptively determines the relative contribution of the image and skeleton memories through cross-attention, without introducing any explicit layer-wise fusion schedule or gating mechanism. As analyzed in Section 3.3, the learned attention distribution exhibits a progressive pattern: the early decoder layers mainly attend to visual representations, whereas the contribution of skeleton-based structural cues gradually increases in deeper layers. At the final decision stage, these GCN-derived features provide complementary structural information to compensate for ambiguities in purely visual representations, particularly under human–object occlusion or blurred object boundaries.

### 2.5. Loss Function

During training, bipartite matching between predicted quintuplets and ground-truth annotation is performed using the Hungarian algorithm [28]. This ensures consistent optimization of human, object, and interaction confidences, as well as bounding box refinements, as illustrated in Figure 5.

Decoder outputs are first parsed by an MLP into interaction instances, which are then matched with ground-truth triplets. The matching loss combines classification and bounding box regression terms:

(7) $$L_{box}=|x_{i}-x_{ij}\left|+\right|y_{i}-y_{ij}\left|+\right|h_{i}-h_{ij}\left|+\right|w_{i}-w_{ij}|,$$

where i∈{h,o}, j∈{1,2}.

The overall matching loss is defined as

(8) $$L_{match}\left(g_{i},P_{i}\right)=\beta_{1}\sum_{j\in \{h,o,r\}}\alpha_{j}L_{cls}^{j}+\beta_{2}\sum_{j\in \{h,o,r\}}L_{box}^{k},$$

where $\alpha_{j}$ denotes class-specific weights (human, object, interaction), $\beta_{1}$ and $\beta_{2}$ balance classification and regression terms, $g_{i}$ are the ground-truth labels, while others are treated as background. The network is trained end-to-end via backpropagation to progressively improve HOI detection accuracy.

### 2.6. Experimental Setup

We conduct experiments on the V-COCO dataset, a subset of MS-COCO designed for HOI detection. It contains 5400 training and 4946 test images, annotated with 29 action categories (5 of which are human-only). Human skeletons (18 keypoints) are extracted with AlphaPose [29], while ground-truth objects are used for training and YOLOv8s detections for testing.

Images are normalized before input. We use ResNet-50 [30] as the CNN backbone, and a 6-layer encoder–decoder Transformer initialized from DETR. The GCN branch is randomly initialized. AdamW is used with learning rates of 1 × 10^−5^ for CNN and 1 × 10^−4^ for Transformer. Models are trained for 250 epochs. Unless otherwise specified, the final SET-HOI model uses $\alpha_{h}=1$, $\alpha_{o}=1$, $\alpha_{r}=1$, which achieves the best performance according to the weight ablation study.

## 3. Results and Discussion

### 3.1. Comparative Study

We reproduce HOI Transformer under the same settings and compare with SET-HOI. As shown in Table 1, SET-HOI achieves 51.6% mAP on V-COCO and is further compared with representative Transformer-based HOI detectors, including HOTR, QPIC, and GEN-VLKT. These methods adopt different strategies and computational trade-offs for improving interaction recognition. In particular, GEN-VLKT enhances interaction understanding by transferring visual-linguistic knowledge from CLIP, whereas SET-HOI focuses on introducing explicit skeleton–object structural priors through a lightweight GCN branch. Compared with our reproduced HOI Transformer baseline, SET-HOI improves the mAP from 48.9% to 51.6%, corresponding to an absolute gain of 2.7 mAP points.

To further evaluate the computational efficiency for real-time embodied AI and biomimetic robotic applications, we compare SET-HOI with representative Transformer-based HOI detectors in terms of model parameters, FLOPs, latency, and throughput, as summarized in Table 2. SET-HOI contains only 41.73 M parameters and requires 113.70 G FLOPs, while achieving a core inference latency of 15.12 ms (66.13 FPS). Compared with HOTR, SET-HOI reduces the number of parameters from 51.18 M to 41.73 M and achieves a higher reported throughput (66.13 FPS versus 16.39 FPS). Compared with QPIC, SET-HOI also exhibits substantially higher reported inference throughput (66.13 FPS versus 13.78 FPS).

More importantly, compared with the HOI Transformer baseline, the proposed skeleton-GCN branch introduces only 0.064 M additional parameters (+0.15%) and 0.017 G GLOPs (+0.015%), while improving the detection performance by 2.7 mAP points. The core model latency remains nearly unchanged (15.12 ms versus 15.30 ms), demonstrating that explicit structural reasoning introduces negligible computational overhead.

For completeness, SET-HOI requires front-end pose estimation and object detection to construct the skeleton–object graph. When the AlphaPose and YOLOv8s preprocessing stages are included, the complete end-to-end pipeline requires approximately 26.12 ms per image, corresponding to 38.28 FPS. Although this preprocessing introduces additional latency, the complete system still maintains real-time throughput above 30 FPS.

### 3.2. Ablation Study

#### 3.2.1. Graph Ablation Study

To test the role of structured skeletal modeling, we compare three variants: visual-only, skeleton heatmap input, and skeleton–object graph (SET-HOI). Table 3 shows that the heatmap representation degrades performance, while the graph-based encoding significantly boosts detection, especially for fine-grained actions (e.g., ride-instr, ski-instr, read-obj).

The reported ablation results are obtained from single training runs and are intended as controlled empirical comparisons; no formal statistical significance test is performed.

To reduce the computational cost of repeated ablation experiments, all three variants in Table 3 were trained using the same reduced subset of the V-COCO training set. The reported AP values are therefore intended for controlled relative comparison among different skeleton representations and are not directly comparable with the 51.6% mAP of the final SET-HOI model trained on the full training set.

#### 3.2.2. Weight Ablation Study

To investigate the dependency of the model on the loss function, we conducted a weight ablation study. In human–object interaction (HOI) detection tasks, the human always appears in every HOI instance. Therefore, we can assume that human classification is the most fundamental task and set its weight parameter to $\alpha_{h}=1$. The weights for object classification and interaction classification are denoted as $\alpha_{o}$ and $\alpha_{r}$, respectively. In the model using only the visual branch, the best performance was achieved with $\alpha_{o}=1,\;\alpha_{r}=2$ [4]. However, as shown in Table 4, the SET-HOI model achieved the best detection performance with $\alpha_{o}=1,\;\alpha_{r}=1$.

This result indicates that the introduction of the skeleton-GCN branch provides strong and direct prior pose information for interaction recognition, fundamentally changing the flow of information in the model. In the visual-only model, interaction information must be inferred entirely from static features in an implicit manner, a process that is highly uncertain and difficult. Therefore, a larger interaction classification loss weight $\alpha_{r}=2$ is needed to reinforce the supervisory signal. In contrast, SET-HOI explicitly processes human and object keypoints through the GCN branch, effectively relieving the visual branch from the burden of interaction recognition. As a result, the learning difficulty of the three subtasks—human, object, and interaction—becomes more balanced, and a lower interaction loss weight $\alpha_{r}=1$ better facilitates the deep fusion of visual and pose information, thereby achieving the best performance.

In addition, we observed that when $\alpha_{o}=1,\alpha_{r}=0.5$, the model’s accuracy actually decreases. This suggests that when the interaction loss is set too low, the model pays insufficient attention to the interaction task and fails to fully exploit the information provided by the skeleton-GCN branch. This further demonstrates the effectiveness and necessity of SET-HOI’s multi-modal architecture: while the GCN branch provides strong support to the model, it does not completely replace the importance of visual contextual information.

### 3.3. Visualization Analysis

To further analyze how skeleton information contributes to interaction recognition, we examined the attention distribution within the Transformer decoder. During testing, we visualized the cross-attention of each layer in the decoder and calculated the attention weights contributed by the GCN branch, as shown in Figure 6. In the early decoder layers, the attention assigned to the skeleton branch remains relatively small, with visual features contributing the majority of the cross-attention response. As the decoder becomes deeper, the contribution of skeleton-based structural cues gradually increases. This emergent attention pattern indicates that the network learns to first establish coarse interaction context from visual features and subsequently incorporates explicit structural priors for refined interaction reasoning. As illustrated in the detection results of Figure 7, the SET-HOI model is able to maintain reliable performance when handling images with blurred object boundaries or severe occlusion of human and objects, thereby validating its robustness in complex scenarios.

### 3.4. Limitations and Failure Case Analysis

As reported in Table 2, while SET-HOI achieves superior overall performance (+2.7% mAP), a slight performance dip is observed in highly dynamic categories like *hit-instr* and *kick-obj*. This limitation stems from upstream pose-estimation constraints under rapid motion and severe body contortion. High-speed interactions frequently induce motion blur and extreme joint self-occlusion, leading to localized keypoint localization errors from the front-end pose estimator. Since the GCN propagates features along explicit spatial edges, large keypoint displacements introduce localized structural noise into the skeleton graph memory. Similarly, when object boundaries are severely blurred or occluded, inaccurate object detections may lead to unreliable bounding-box corner nodes or missing human–object candidate pairs, thereby weakening the structural cues provided by the skeleton branch. Therefore, the current framework remains dependent on the quality of the upstream pose-estimation and object-detection modules. In future work, incorporating temporal smoothing or uncertainty-guided graph filtering will mitigate this sensitivity to noisy pose inputs.

## 4. Conclusions

In this paper, we presented SET-HOI, a novel dual-branch skeleton-enhanced Transformer architecture designed for real-time human–object interaction detection. By incorporating a lightweight GCN branch to model explicit joint-object kinematic topologies and concatenating these representations into a unified Structure-Enhanced Memory, SET-HOI effectively compensates for the limitations of pure visual features under severe human occlusion and ambiguous object boundaries. Extensive experiments on the V-COCO dataset demonstrate that SET-HOI achieves a competitive performance of **51.6% mAP**, yielding an absolute improvement of 2.7 mAP points over standard single-branch HOI baselines. Importantly, the model achieves a core inference latency of **15.12 ms** with negligible extra FLOPs (+0.017 G), supporting an end-to-end processing throughput of **38.28 FPS**.

This ultra-low computational footprint and robust topological reasoning pave the way for seamless integration into real-time Embodied AI frameworks and robotic control systems. In future work, we plan to extend SET-HOI to dynamic video-based interaction sequences and integrate uncertainty-guided graph filtering to further enhance robustness in high-speed, noisy manipulation environments.

## Figures and Tables

**Figure 1:**
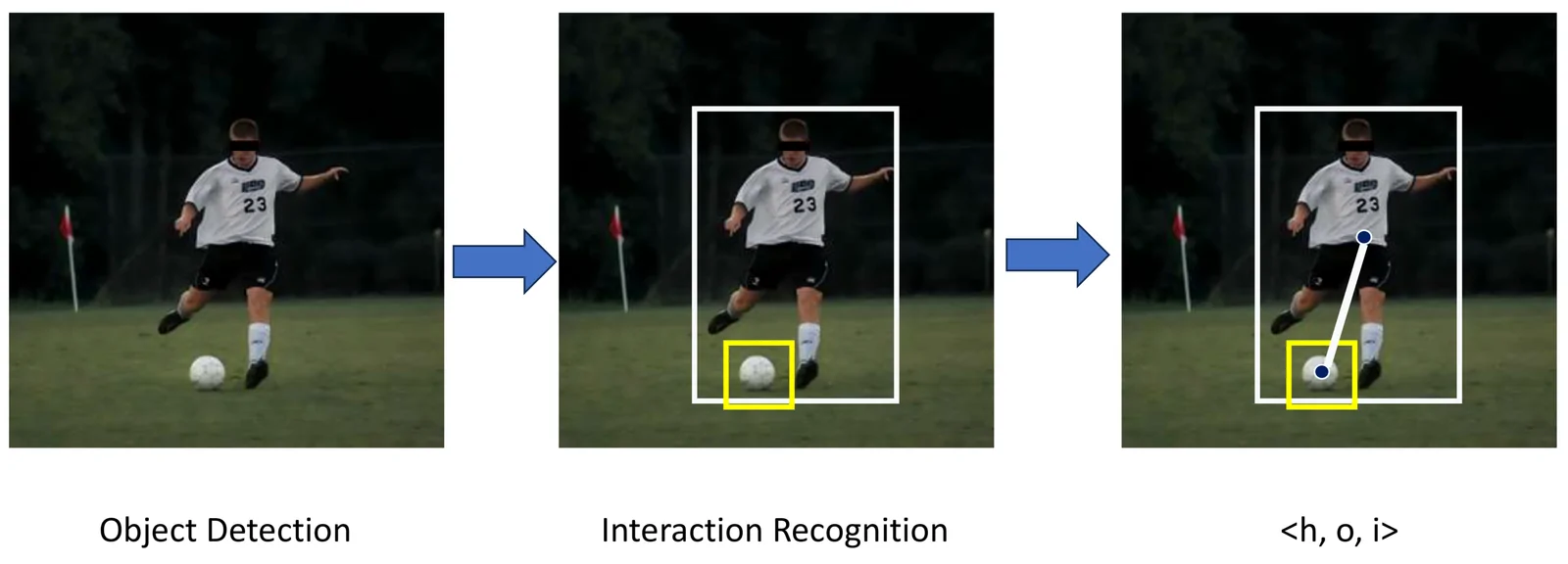
Diagram of two-stage HOI detection [4].

**Figure 2:**
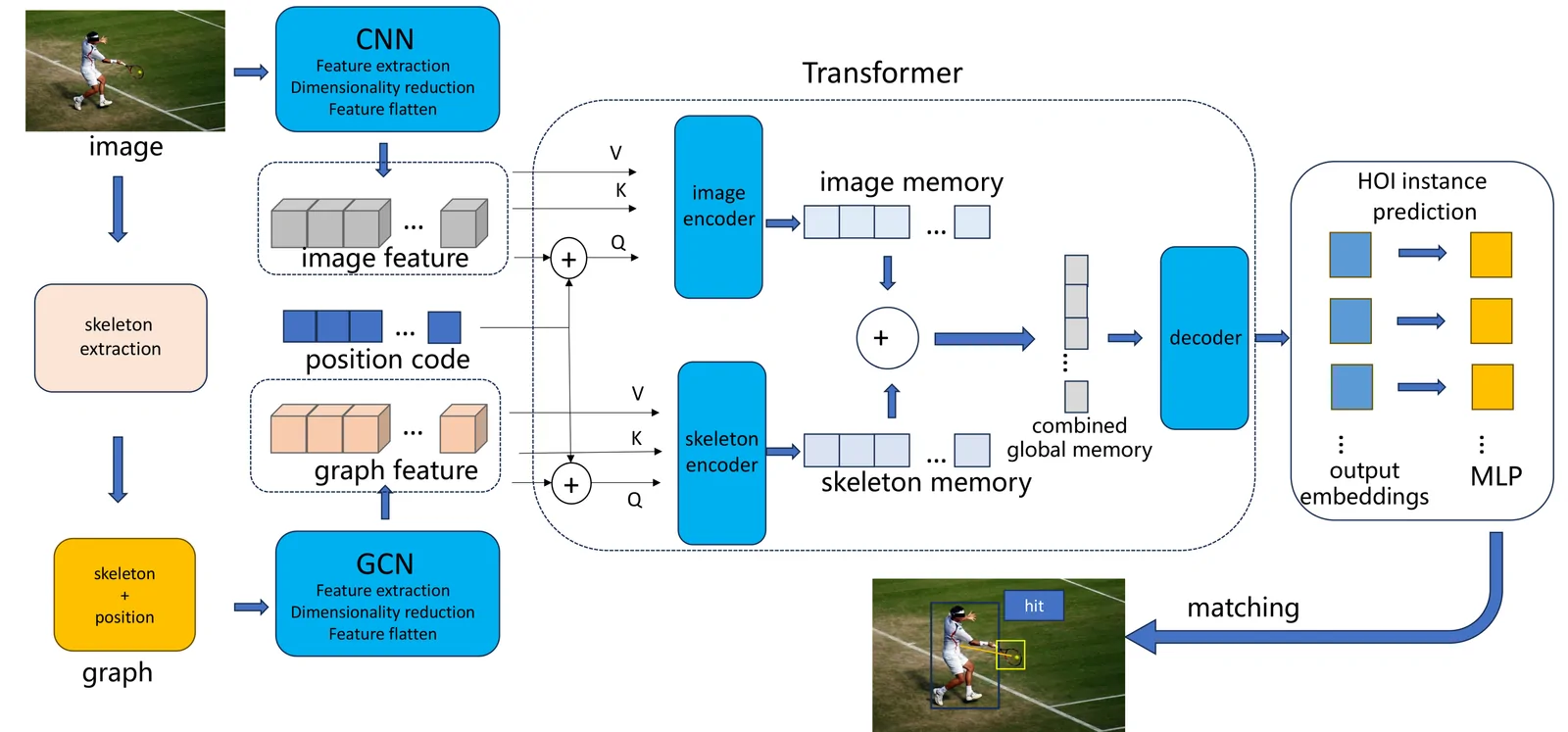
Proposed dual-branch model:SET-HOI. (1) The image memory is obtained through a CNN and image encoder. (2) Meanwhile, the image is fed to a pose-estimation model and an object-detection model to extract skeleton and position graphs, which are the source of skeleton memory. (3) Image memory and skeleton memory are combined and fed to the decoder to obtain the prediction of HOI instances.

**Figure 3:**
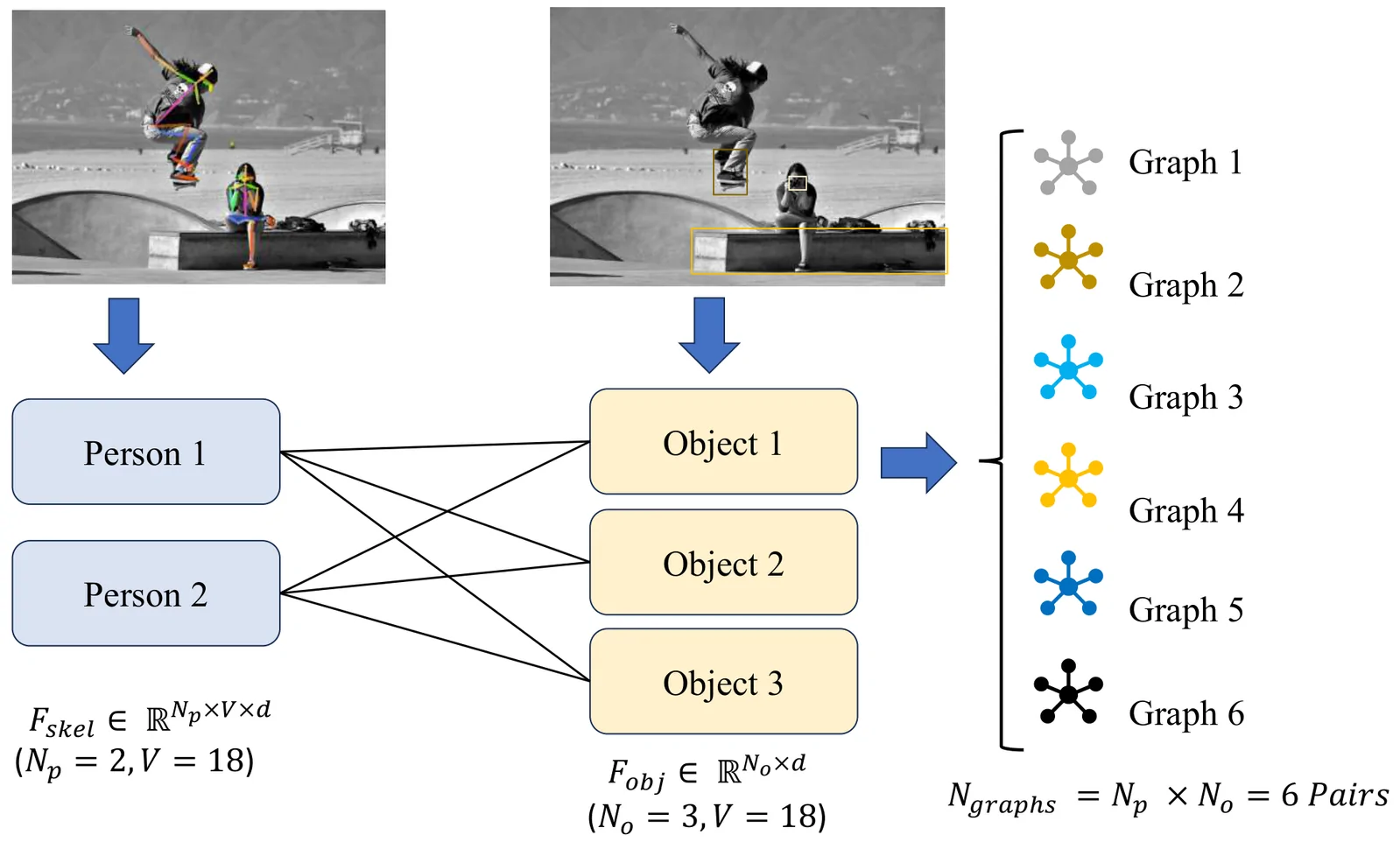
Overview of the full bipartite graph construction. Given $N_{p}$ human candidates ($F_{\mathrm{skel}}\in R^{N_{p}\times V\times d}$) and $N_{o}$ object candidates ($F_{\mathrm{obj}}\in R^{N_{o}\times d}$), the module constructs $N_{\mathrm{graphs}}=N_{p}\times N_{o}$ candidate interaction subgraphs (e.g., $N_{\mathrm{graphs}}=6$ pairs for $N_{p}=2,N_{o}=3$), ensuring explicit topological modeling for every candidate pair. The anatomical adjacency follows the fixed 18-keypoint AlphaPose topology, including facial, shoulder–arm, hip–leg, shoulder–hip, inter-hip, and nose-to-head-top connections. The two diagonal object corner nodes are mutually connected, and each of them is densely connected to all 18 skeletal nodes. Therefore, the graph jointly preserves local anatomical structure and global human–object spatial relationships. All graph edges are fixed and are not dynamically learned.

**Figure 4:**
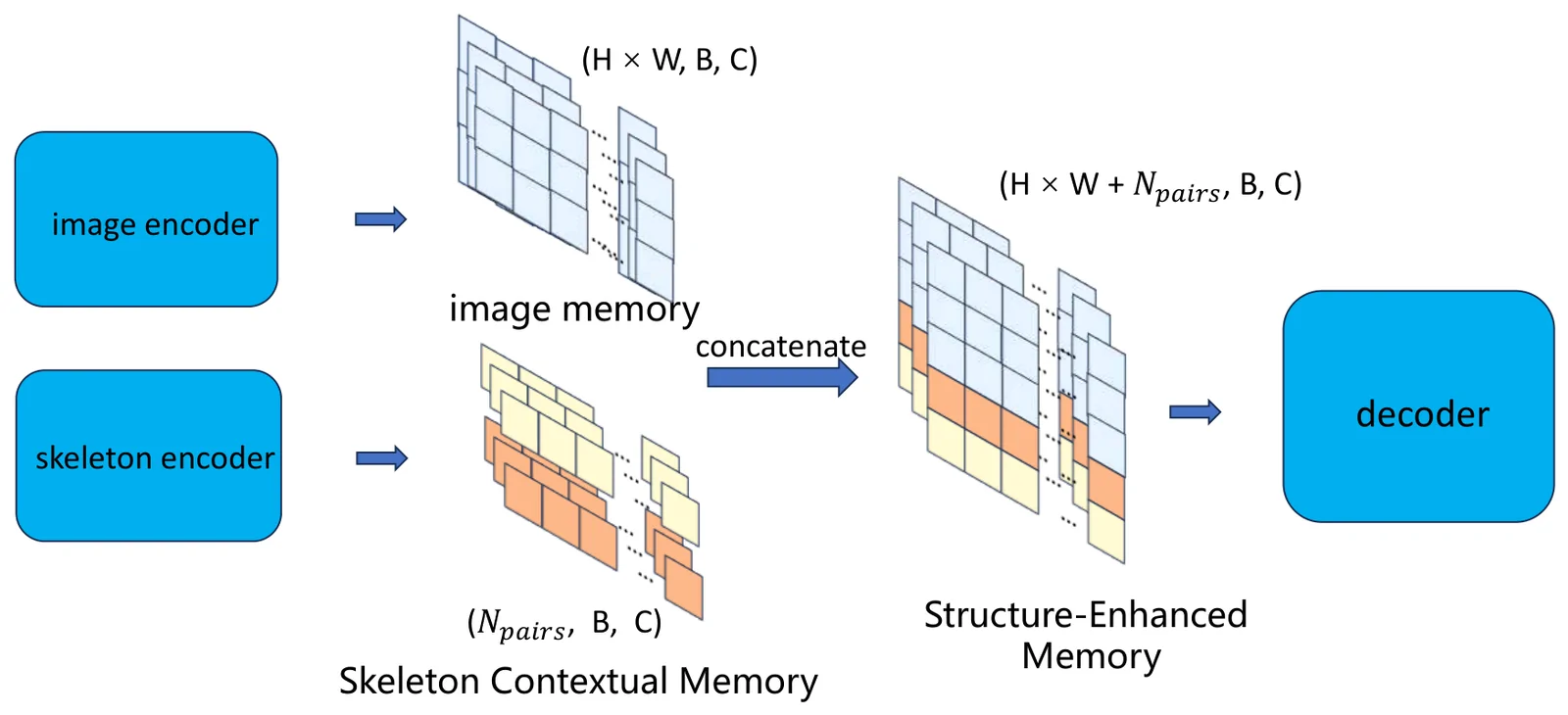
Architecture of the parallel memory fusion module. The image encoder produces the image memory $M_{\mathrm{img}}\in R^{(H\times W)\times B\times C}$, while the skeleton encoder transforms the pair-wise GCN graph embeddings into the skeleton contextual memory $M_{\mathrm{skel}}\in R^{N_{\mathrm{pairs}}\times B\times C}$. The two memories are concatenated along the sequence dimension to form the unified Structure-Enhanced Memory $M_{\mathrm{struct}}\in R^{(H\times W+N_{\mathrm{pairs}})\times B\times C}$, which is subsequently fed into the Transformer decoder for interaction reasoning.

**Figure 5:**
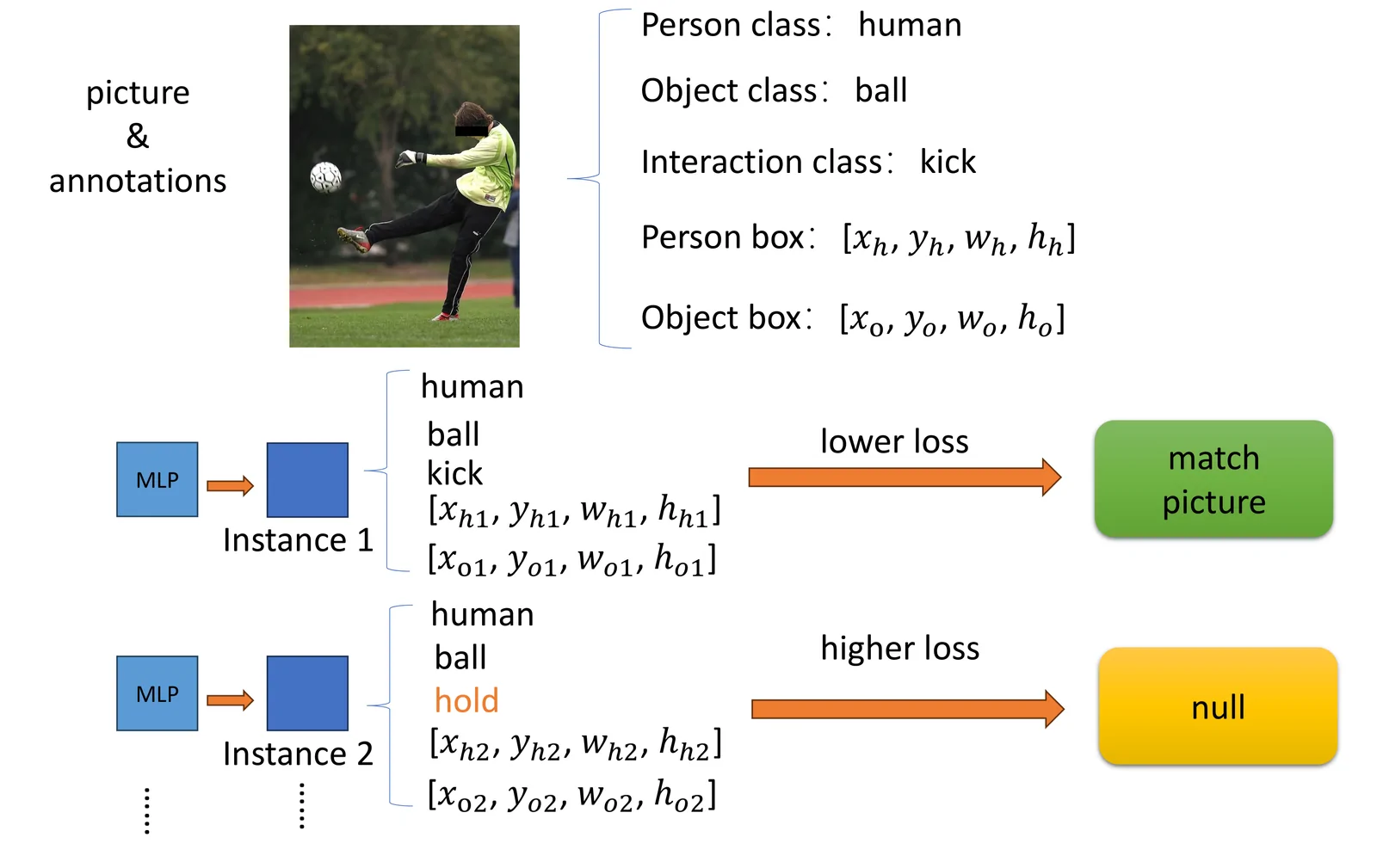
Example of ground-truth matching. Each image results in multiple instances, with labels assigned based on the result value of the loss function.

**Figure 6:**
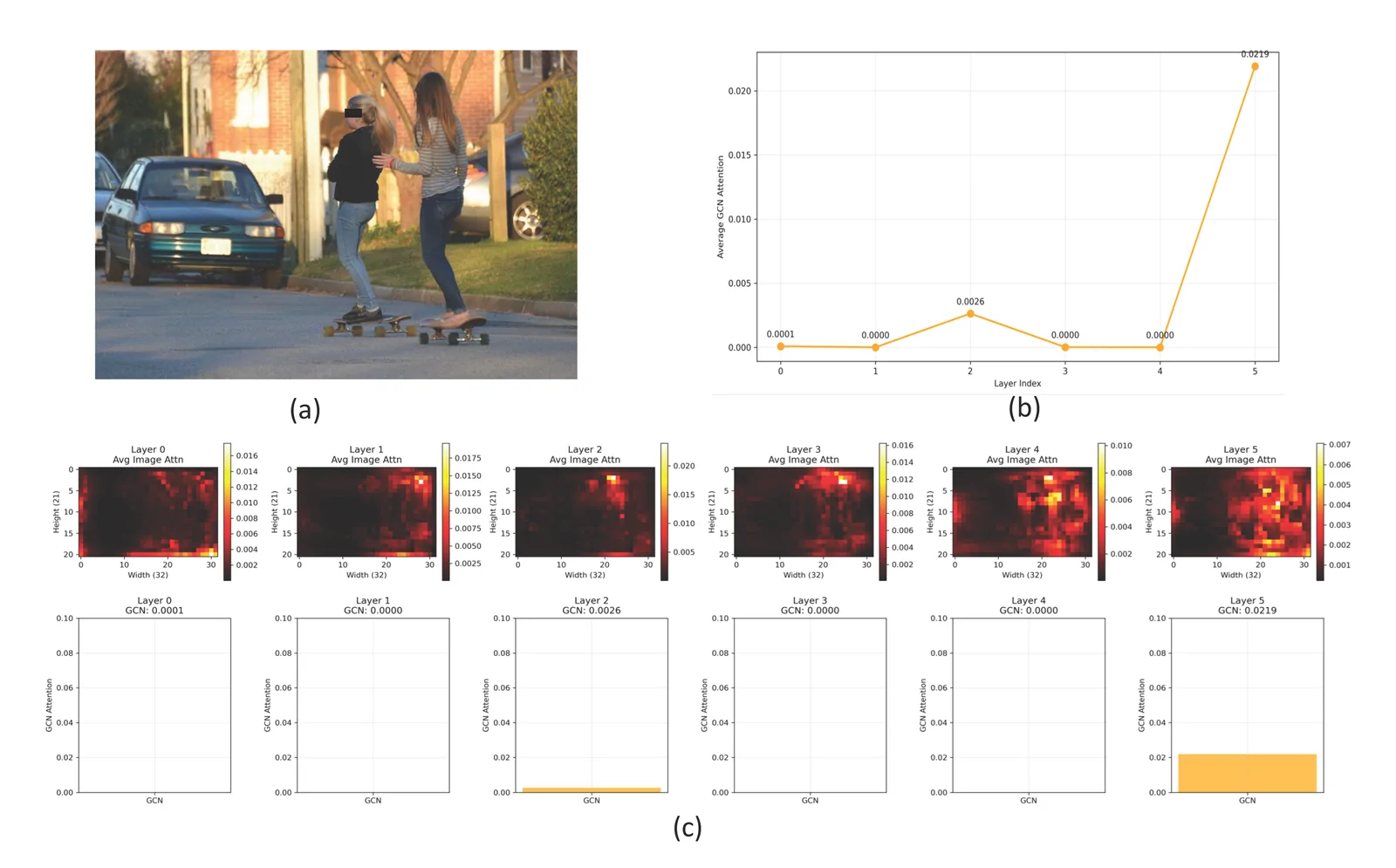
Visualization of cross-attention and the distribution of GCN attention. (**a**) The origin image, (**b**) indicates the proportion of GCN attention in each layer of the decoder. (**c**) Visualization of cross-attention.

**Figure 7:**
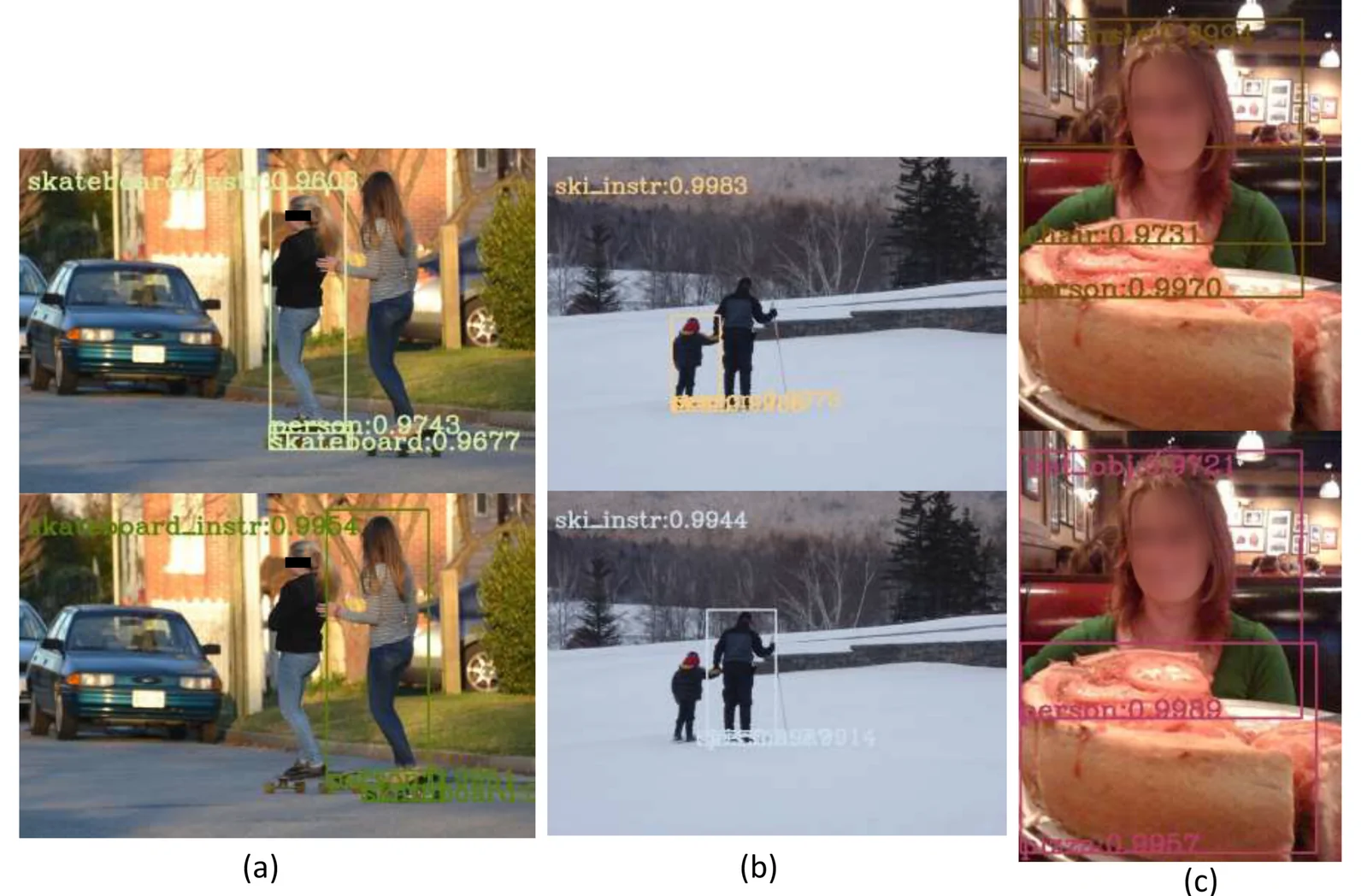
Result of HOI detection by SET-HOI. (**a**) The detection of the image in Figure 6, (**b**) image with blurred object boundaries, (**c**) image with occlusion of both human and object.

**Table 1:** Comparison with other models.

Methods	Backbone	mAP (%)
VSRL [31]	ResNet-50-FPN	31.8
GPNN [26]	ResNet-50	44.0
RPNN [32]	ResNet-50	47.5
PastaNet [33]	ResNet-50	48.9
HOTR [14]	ResNet-50	55.2
QPIC [13]	ResNet-50	58.8
GEN-VLKT [17]	ResNet-50	62.41
UnionDet [34]	ResNet-50-FPN	47.5
HOI Transformer [4]	ResNet-50	48.9
**Ours**	**ResNet-50**	**51.6**

Boldface identifies the results of the proposed SET-HOI method.

**Table 2:** Computational complexity and real-time performance comparison on the V-COCO dataset.

Model/Pipeline	mAP (%)	Params (M)	FLOPs (G)	Latency (ms)	Total FPS
HOTR	55.2	51.18	-	61.00	16.39 *
QPIC	58.8	-	-	-	13.78
HOI Transformer	48.9	41.67	113.68	15.30	65.34
SET-HOI	51.6	41.73	113.70	15.12	66.13

* The HOTR FPS is converted from the inference latency reported in its original publication.

**Table 3:** Comparison.

Interaction	Image Branch Only	Skeleton Heatmap	Ours
hold-obj	34.73	22.54	37.45
ride-instr	61.97	45.24	65.61
hit-instr	67.64	48.76	65.04
eat-obj	40.61	29.43	43.02
jump-instr	50.58	39.94	50.16
lay-instr	32.23	24.52	34.78
carry-obj	34.68	21.37	43.20
throw-obj	43.83	33.82	44.91
catch-obj	48.49	35.13	45.74
cut-obj	41.60	27.39	41.89
ski-instr	36.30	26.86	39.55
skateboard-instr	81.33	61.62	82.59
drink-instr	39.48	24.49	57.81
kick-obj	76.48	73.17	73.79
read-obj	27.74	16.45	39.01
snowboard-instr	71.68	59.96	69.26
…	…	…	…
**Average AP**	**43.36**	**31.48**	**45.19**

The reported “Average AP” is computed across all 24 human–object interaction categories in V-COCO (excluding 5 human-only classes). And the boldface identifies the summary row reporting the average AP of each model variant across all 24 HOI categories.

**Table 4:** Effect of object and interaction classification loss weights on V-COCO mAP.

$\alpha_{o}$	$\alpha_{r}$	mAP (%)
1	2	50.3
1	1	51.6
1	0.5	51.0

## Data Availability

The dataset we use in this study is the V-COCO Human–Object Interaction dataset. This dataset can be downloaded via https://cocodataset.org/#download (accessed on 1 March 2026).
